# Allosteric coupling between Mn^2+^ and dsDNA controls the catalytic efficiency and fidelity of cGAS

**DOI:** 10.1093/nar/gkaa084

**Published:** 2020-03-14

**Authors:** Richard M Hooy, Guido Massaccesi, Kimberly E Rousseau, Michael A Chattergoon, Jungsan Sohn

**Affiliations:** 1 Department of Biophysics and Biophysical Chemistry, Johns Hopkins University School of Medicine, Baltimore, MD 21205, USA; 2 Division of Infectious Diseases, Johns Hopkins University School of Medicine, Baltimore, MD 21205, USA

## Abstract

Cyclic-G/AMP (cGAMP) synthase (cGAS) triggers host innate immune responses against cytosolic double-stranded (ds)DNA arising from genotoxic stress and pathogen invasion. The canonical activation mechanism of cGAS entails dsDNA-binding and dimerization. Here, we report an unexpected activation mechanism of cGAS in which Mn^2+^ activates monomeric cGAS without dsDNA. Importantly, the Mn^2+^-mediated activation positively couples with dsDNA-dependent activation in a concerted manner. Moreover, the positive coupling between Mn^2+^ and dsDNA length-dependent activation requires the cognate ATP/GTP substrate pair, while negative-cooperativity suppresses Mn^2+^ utilization by either ATP or GTP alone. Additionally, while Mn^2+^ accelerates the overall catalytic activity, dsDNA length-dependent dimerization specifically accelerates the cyclization of cGAMP. Together, we demonstrate how the intrinsic allostery of cGAS efficiently yet precisely tunes its activity.

## INTRODUCTION

cGAS is crucial for the host defense against various maladies that give rise to cytosolic dsDNA (e.g. pathogen invasion, genotoxic stress, and dysfunctional mitochondria) (1,2). Upon binding and dimerizing on cytosolic dsDNA, cGAS cyclizes ATP and GTP into cGAMP, a second messenger that activates type-I interferon (IFN-I)-mediated innate immune responses via Stimulator of Interferon Genes (STING) (2,3). cGAS is essential for the host defense against numerous pathogens (1,4,5), regulates tumorigenesis (6), and is involved in autoimmunity (7–10).

It is increasingly appreciated that cGAS dynamically tunes the intensity and duration of the IFN-I activity according to the severity of stress, consequently eliciting context-dependent innate immune responses (e.g. autophagy, cellular senescence, and apoptosis) (1,2,11–14). It was recently identified that the length of cytosolic dsDNA is a key measure for cGAS to gauge stress levels (15,16): long stretches of naked dsDNA (≥ 50 base-pairs (bps)) signify major maladies such as the presence of viral genomes or mislocalized mitochondrial dsDNA, while shorter fragments indicate minor DNA damage or degraded viral genomes (15,16). How does dsDNA length regulate cGAS activity? Resting cGAS is an inactive monomer and dimerization is required for activation in addition to dsDNA binding (17,18). dsDNA length dictates the distribution of dsDNA-bound cGAS monomers and dimers, thereby grading the signaling activity (19). Importantly, such an allosteric mechanism allows dsDNA length-dependence to be conditional: substrate binding and elevated cGAS concentrations also induce dimerization, thereby reducing the dependence on dsDNA length (19).

Still, major questions remain regarding how cGAS is regulated. First, it was recently reported that cytosolic Mn^2+^ originating from damaged organelles potentiates the dsDNA sensing activity of cGAS (20). In fact, Mn^2+^ was crucial for cGAS-mediated host innate immune responses against DNA viruses (20). Nevertheless, how Mn^2+^ regulates GAS activity remains unknown. Second, little is known about how cGAS retains substrate specificity and signal fidelity (3,21,22). The catalytic core of cGAS contains a polymerase (pol)-β-type nucleotidyl-transferase (NTase) domain, which is inherently promiscuous (23–25). Although ATP/GTP (1ATP:1GTP) is the cognate substrate pair, cGAS can bind and perform chemistry on non-cognate substrate pairs (e.g. ATP/ATP). Such ‘off-pathway’ interactions can be deleterious by not only competing against cognate substrates, but also generating dinucleotides that do not activate STING (3,21,22,26). Moreover, this issue can also be exacerbated by multiple factors: (**a**) ATP outnumbers GTP by at least 4-fold *in vivo* (27) and both NTPs, especially ATP, are present at saturating concentrations for cGAS ([ATP] and [GTP] > K_M_ for cGAS) (8,19,22,28). Thus, noncognate ATP/ATP is likely the most abundant substrate pair for cGAS. How cGAS overcomes this issue and generates cGAMP in high fidelity remains unknown. (**b**) Although Mn^2+^ sometimes enhances the catalytic activity of other cGAS-like NTases by substituting for Mg^2+^, its primary effect is increased substrate promiscuity (23–25). In fact, Mn^2+^ is considered mutagenic for pol-β-type NTases as it preferentially enhances the affinity for noncognate NTPs (23–25). Currently, how cGAS could compensate for the potentially deleterious effect of Mn^2+^ remains unknown. (**c**) Human cGAS is a poor cyclase (3,8,22,28). cGAS first generates 2′-GTP to 5′-ATP-linked linear pppGpA from ATP/GTP, which dissociates and rebinds to be cyclized into 2′-5′ 3′-5 linked cGAMP (3,22) (simply ‘cGAMP’ hereafter). Because of low cyclization efficiency, the pppGpA intermediate accumulates much more rapidly than cGAMP (3,22). This could impinge on promptly mounting host responses, as the intermediate cannot activate STING or even be degraded rapidly. Again, how cGAS might address this issue remains unknown. Here, we unravel an unexpected role of allostery in resolving all these compounding issues.

We find that Mn^2+^ activates human cGAS without dsDNA and also positively couples with dsDNA-dependent activation, allowing Mn^2+^ to dampen dsDNA length-dependence in activating cGAS both *in vitro* and in macrophages. cGAS preferentially utilizes Mn^2+^ over Mg^2+^ for its NTase activity, and Mg^2+^ facilitates Mn^2+^ utilization instead of competing against it. Moreover, physiological amounts of Mn^2+^ can activate cGAS mutants defective in dsDNA/dimerization-dependent activation both *in vitro* and in cells, indicating that Mn^2+^ activates monomeric cGAS. The NTase activity with cognate ATP/GTP vs. noncognate ATP/ATP or GTP/GTP is similar when cGAS is bound to short dsDNA; however, longer dsDNA increases the activity only toward ATP/GTP. Likewise, although Mn^2+^ accelerates the NTase activity of cGAS toward noncognate substrates, ATP/GTP benefits most. Furthermore, ATP/GTP is required for the synergy between Mn^2+^ and dsDNA binding, while dsDNA binding invokes negative-cooperativity between Mn^2+^ and noncognate substrates. Finally, Mn^2+^ accelerates the overall catalytic activity of cGAS; however, dsDNA length-dependent dimerization specifically accelerates the rate-determining cyclization, minimizing the formation of off-pathway products even with excess ATP. Together, we demonstrate how allostery linking Mn^2+^- and dsDNA-binding maximizes the efficiency and activity of cGAS.

## MATERIALS AND METHODS

### Protein expression and purification

Human cGAS (encoding 2–522, cGAS^FL^ or 157–522, cGAS^cat^) was cloned into a pET28b vector (Novagen) in-frame with an N-terminal 6xHis-MBP-tag and a TEV protease cleavage site. Plasmids encoding wild-type and mutant cGAS constructs were transformed in to *E. coli* BL21-Rosetta 2 DE3 cells and expressed via IPTG as described in ref. (19). Recombinant cGAS was then purified as described in (19), concentrated, and stored in −80°C.

### Reagents

dsDNAs longer than 90-bp were PCR amplified from the human DDX41 gene and agarose gel purified. dsDNAs shorter than 90-bp were purchased from Integrated DNA Technology. ATP and GTP were hydrated separately in 20 mM HEPES buffer and adjusted to pH 7.4 with NaOH.

### Biochemical assays

All experiments were performed at least three times as described in before (19,29). Shown Figures are either representative of these independent experiments or averages with standard deviations (SD). The fits to data (the Hill form of the Michaelis-Menten equation or the binding isotherm) were generated using Kaleidagraph (Synergy Software). All reactions were performed under 25 mM Tris acetate pH 7.4, 125 mM potassium acetate pH 7.4, 0.5 mM TCEP, 5% glycerol and indicated concentrations of MgCl_2_ and/or MnCl_2_ at 25 ± 2°C. cGAS concentrations are noted in Figure Legends. To avoid titrating Mn^2+^ against free NTPs and to mimic the *in vivo* environment where Mg^2+^ is present in large excess (20,30), each NTP was pre-mixed with 1 molar equivalent of MgCl_2_, with the exception of those experiments that were conducted in the complete absence of MgCl_2_, in which case the NTPs were pre-mixed with equimolar MnCl_2_ instead. We used as low as 6 μM MnCl_2_ in our experiments to reflect the physiological amount (≤ 53 μM; (20,31,32)).

### Analytical fractionation of cGAS reactions by HPLC

40 μl reactions containing cGAS, NTPs, dsDNA (where indicated), MgCl_2_ and/or MnCl_2_ were incubated in reaction buffer for the indicated time. Reactions were quenched with 25 mM EDTA, diluted to 80 μl with water and filtered with 3 or 10 KDCO spin columns (Amicon). An aliquot of the flow-through was mixed 1:1 with HPLC buffer A (see below) and fractionated. HPLC fractionation was performed on the Agilent Technologies 120 Infinity II system using a Poroshell EC-C18 column (2.7μm; 4.6 × 100mm). Reaction products were fractionated by the following scheme: 0% B from 0–1min, linear increase to 50% B from 1–27 min, linear increase to 100% B from 27–28 min. Buffer A: 100 mM potassium phosphate monobasic, 5 mM TBA, final pH 6.0; Buffer B: 100 mM potassium phosphate monobasic, 5 mM TBA, final pH 6.0, 30% acetonitrile. Buffers were filtered and degassed. Integrated peak intensities were calculated by the Agilent Technologies ChemStation software. Synthetic and purified compounds were used as standards for peak identification.

### Human cell line culture

THP1-Dual cells (InvivoGen) were passaged and cultured in RPMI 1640 media containing 2 mM L-glutamine, 25 mM HEPES at pH 7.4, 10% heat-inactivated fetal bovine serum (30 min at 56°C), 100 μg/ml Normocin™, Pen-Strep (100 U/ml-100 μg/ml), Blasticidin (10ug/mL) and Zeocin (100ug/ml). HEK293T/17 cells (ATCC) were passaged and cultured in DMEM media containing 2 mM L-glutamine, 1 mM sodium pyruvate and 10% heat-inactivated fetal bovine serum (30 min at 56°C).

### Measuring cGAS activity in THP-1 cells

THP1-Dual and THP1-Dual *cGAS-KO* reporter monocytes were differentiated into macrophages with 50 nM PMA for 16 hours at 37°C and 5% CO_2_. The PMA containing media was replaced by fresh media and cells were incubated an additional 48 hours at 37°C 5% CO_2._ The media was again replaced with fresh media or media plus MgCl_2_ or MnCl_2_ and incubated for 2 hrs at 37°C and 5% CO_2_. Cells were then transfected with various lengths of dsDNA (1.67 μg/ml final) using Lipofectamine LTX containing OptiMEM media. Cells were incubated an additional 24 hours at 37°C and 5% CO_2_ before supernatants were collected and Lucia production was determined using the Luciferase Reporter Assay System (Promega).

### Measuring cGAS activity in HEK293T cells using dual-luciferase reporter

HEK293T cells were pre-incubated for 2 hours at 37°C and 5% CO_2_ with growth media, or media supplemented with 50 μM of MnCl_2_ or MgCl_2_. 10 or 50 ng of pCMV plasmids encoding empty vector or various cGAS^FL^ variants were then transfected using Lipofectamine LTX (Invitrogen), along with 5 ng of Renilla Luciferase plasmid, 10 ng of plasmid encoding human STING, and 10 ng of plasmid encoding Firefly Luciferase under an IFN-Iβ promoter. Transfected cells were incubated overnight at 37°C and 5% CO_2,_ washed once with PBS and lysed. Lysates were analyzed for Firefly Luciferase and Renilla Luciferase activities independently using the Dual-Luciferase Reporter Assay System (Promega). Data shown is normalized as the ratio of Firefly output divided by log_10_ Renilla output of individual wells.

## RESULTS

### Mn^2+^ dampens the dependence on dsDNA length in activating cGAS

**Figure 1.**
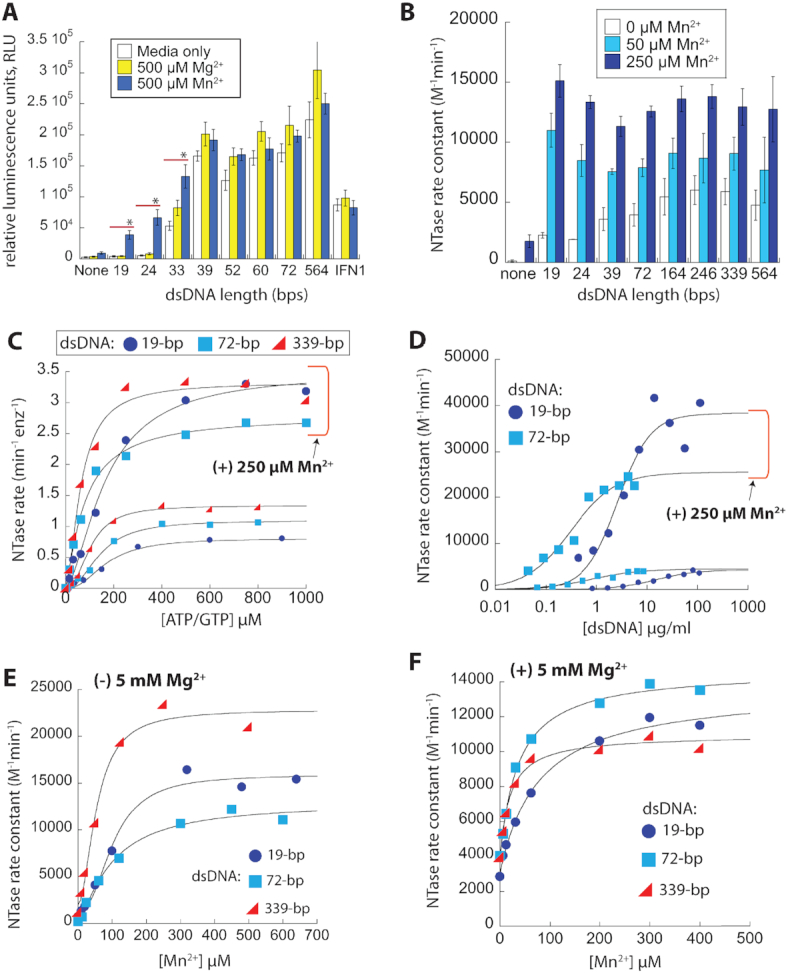
Mn^2+^ dampens the dsDNA length-dependent activation of cGAS. (**A**) dsDNA length-dependent signaling in THP1 Dual cells in the presence of media only, media plus MgCl_2_ or MnCl_2_. *P < 0.05. *n* = 3, ± standard deviation (SD). (**B**) The NTase activities of 25 nM cGAS^FL^ in the presence of 5 mM MgCl_2_, increasing [Mn^2+^], and saturating amounts of various dsDNA lengths. [ATP/GTP] = *K*_M_ for each dsDNA length as determined in (19), saturating [dsDNA] = 10-fold higher than their apparent affinity (*K*_act_) as determined in (19). *n* ≥ 3, ± SD. (**C**) The NTase activity of 25 nM cGAS^FL^ as a function of [ATP/GTP] in the presence of saturating dsDNA, 5 mM MgCl_2_, and with or without 250 μM MnCl_2_. (**D**) The NTase activity of 25 nM cGAS^cat^ as a function of [dsDNA] in the presence or absence of 250 μM MnCl_2_. 5 mM MgCl_2_, [ATP/GTP] = *K*_M_ for each dsDNA. (E, F) The NTase activity of 25 nM cGAS^FL^ as a function of [MnCl_2_] with different lengths of dsDNA (saturating) in the absence (**E**) or presence (**F**) of 5 mM MgCl_2_. [ATP/GTP] = *K*_M_ for each dsDNA length.

dsDNA length-dependent dimerization is crucial for cGAS activation (15–19). It was reported that cytosolic Mn^2+^ potentiates the dsDNA-sensing activity of cGAS through an unknown mechanism (20). To explore whether Mn^2+^ modulates dsDNA length-dependent activation, we preincubated THP1-Dual reporter cells with Mn^2+^ or Mg^2+^, and transfected dsDNA of various lengths (Figure 1A). Consistent with previous studies (15,16), we observed step-like dsDNA length-dependent luciferase activity without Mn^2+^ (i.e. no significant activity for dsDNA < 33 bps; Figure 1A). With Mn^2+^, dsDNA fragments shorter than 39-bps produced significantly higher IFN-I activity, dampening duplex length-dependence (Figure 1A). By contrast, THP1-Dual *cGAS-KO* cells did not show increased IFN-1 activity upon transfecting dsDNA (Supplementary Figure S1A), indicating that our observations were specific for cGAS.

While investigating the underlying mechanism, we found that Mn^2+^ alone does not enhance the dsDNA binding activity of human full-length cGAS (cGAS^FL^; Supplementary Figure S1B–D and Supplementary Table S1), which prompted us to test whether Mn^2+^ regulates the catalytic activity. Using our PP_i_ase-coupled NTase assay (19), we found that Mn^2+^ drastically increased the maximal dsDNA-induced NTase activity of cGAS^FL^ (*k*_max_) independent of duplex length (Figure 1B), revealing that Mn^2+^ diminishes dsDNA length-dependence by directly enhancing the catalytic activity. Next, we tested whether Mn^2+^ enhances the *K*_M_ or the maximal turnover (*k*_cat_) in the presence of saturating amounts of dsDNA with various lengths (Figure 1C). For all dsDNA lengths, *K*_M_s improved moderately, but *k*_cat_s were consistently and significantly higher (Figure 1C, Supplementary Table S1), indicating that the effect of Mn^2+^ arises from catalysis. Notably, the Hill constants were smaller in the presence of Mn^2+^ (Hill: ∼2 versus ∼1; Figure 1C, Supplementary Table S2), suggesting positive cooperativity between Mn^2+^ and dsDNA-binding in activating cGAS; see (33,34) for reference. To explore this unexpected mechanism, we determined whether Mn^2+^ enhances the apparent affinity of dsDNA (*K*_act_) in the presence of ATP/GTP. Here, we used the catalytic domain (cGAS^cat^) for accurate measurements as it binds dsDNA more weakly than cGAS^FL^ (19). Indeed, cGAS^cat^ showed enhanced *k*_max_s and *K*_act_s for both 19- and 72-bp dsDNA with Mn^2+^ (Figure 1D, Supplementary Table S3). Together, we concluded that Mn^2+^ not only increases the catalytic activity of cGAS, but also allosterically enhances its dsDNA binding activity in conjunction with ATP/GTP.

### cGAS preferentially couples Mn^2+^ to its allosteric activation

We noted that the effect of Mn^2+^ was evident despite excess Mg^2+^ in our assays (Figure 1B–D), suggesting that cGAS preferentially utilizes Mn^2+^ over Mg^2+^. To test this idea, we determined the apparent affinity (EC_50_) of Mg^2+^ and Mn^2+^ in the presence of 339-bp dsDNA (EC_50_: concentration required for 50% activity). The EC_50_ of Mn^2+^ (∼40 μM, Figure 1E) was ∼100-fold tighter compared to Mg^2+^ (∼4 mM, Supplementary Figure S1E), and this preference was maintained across different dsDNA lengths (Figure 1E; Supplementary Tables S4 and S5). Moreover, in contrast to the Hill constant of 1.2 for Mg^2+^, the Hill constants were ∼2 for Mn^2+^ (Figure 1E versus Figure S1E; see also Supplementary Tables S4 and S5), indicating Mn^2+^ can substitute for Mg^2+^ in a positively cooperative manner. In the presence of 5 mM Mg^2+^ (Figure 1F), the EC_50_ of Mn^2+^ improved 2- to 3-fold and the Hill constants also decreased to ∼1, albeit the *k*_max_ decreased ∼ 2-fold (Supplementary Table S5). Considering that cGAS utilizes two divalent metals at the active site (3), our observations suggest that occupying one metal binding site by Mg^2+^ increases the affinity of Mn^2+^ for the second site. The decreased *k*_max_ is also consistent with this idea, as the catalytic activity is supported by both Mg^2+^ and Mn^2+^. Collectively, our observations suggest that free Mg^2+^ in the cytoplasm (∼1 mM (35)) would facilitate Mn^2+^ (≤50 μM (20,31,32)) utilization rather than compete against it (e.g. at 10 μM Mn^2+^, the activity of cGAS with 72-bp dsDNA would be ∼10-fold higher in the presence of Mg^2+^).

### Mn^2+^ activates cGAS without dsDNA and also synergizes with dsDNA-dependent activation

**Figure 2.**
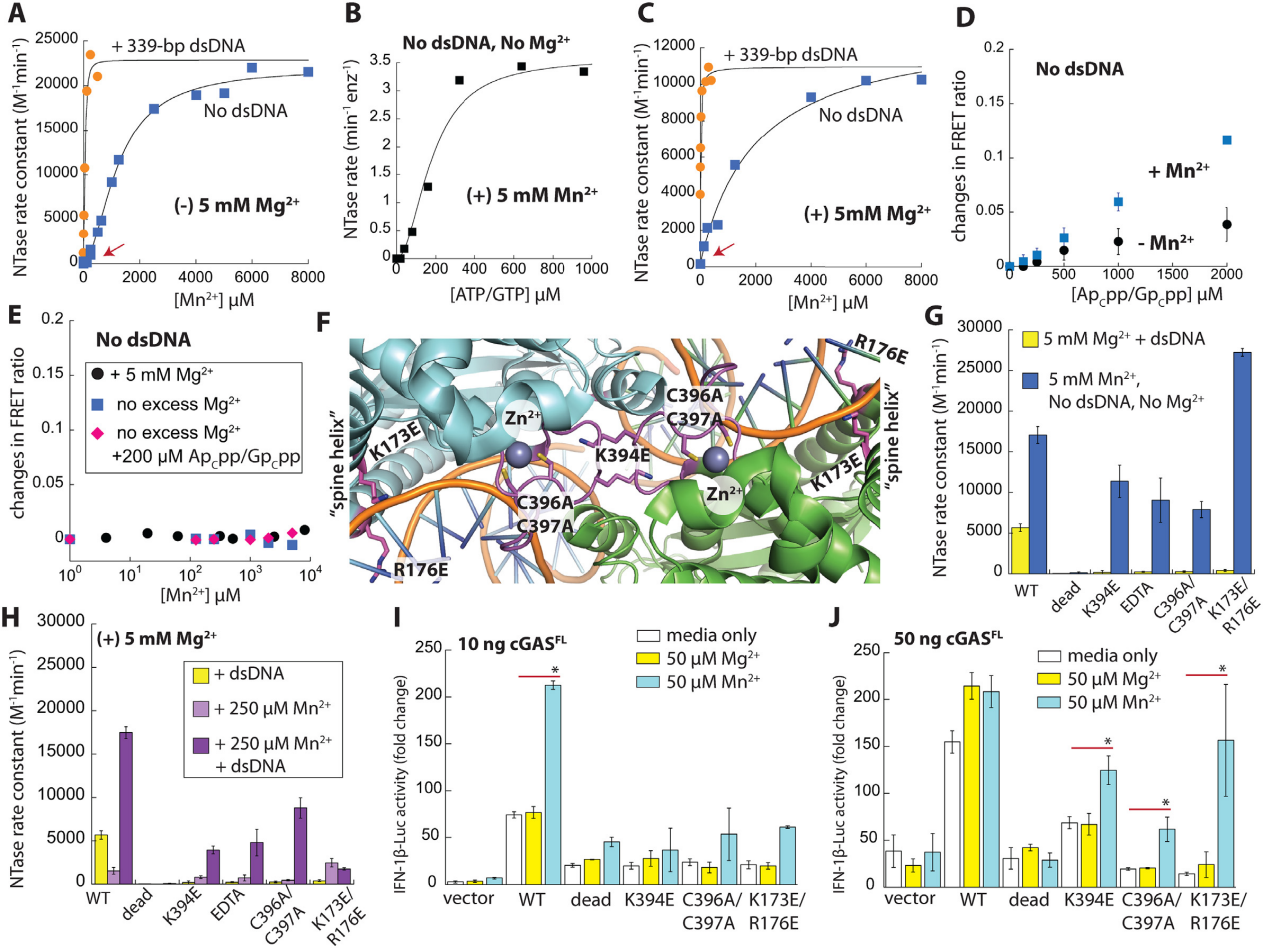
Mn^2+^ activates monomeric cGAS without dsDNA and allosterically couples with dsDNA-mediated activation. (**A**) The NTase activity of 25 nM cGAS^FL^ as a function of [MnCl_2_]. [ATP/GTP] = K_M_. The data in the presence of 339-bp dsDNA from Figure 1E and F are shown for comparison. The red arrows indicate the change in cooperativity. (**B**) The NTase activity of 25 nM cGAS^FL^ as a function of [ATP/GTP] with 5 mM MnCl_2_ and without any MgCl_2_ (‘no Mg^2+^’: each NTP was also pre-bound with equimolar Mn^2+^ instead of Mg^2+^). (**C**) [MnCl_2_] dependent activation of cGASFL, conditions are the same Figure 2A, with the exception of the presence of additional Mg^2+^. (**D**) Changes in FRET ratios between 1:1 TAMRA:Cy5-labeled 20 nM cGAS^FL^ as a function of [Ap_c_pp/Gp_c_pp] in the presence or absence of 5 mM MnCl_2_. Each sample contain 5 mM MgCl_2_. *n* = 2, ± SD. (**E**) Changes in FRET ratios between 1:1 TAMRA:Cy5-labeled 20 nM cGAS^FL^ and 200 μM Ap_c_pp/Gp_c_pp as a function of [MnCl_2_] under various conditions. (**F**) Crystal structure of dsDNA•cGAS^cat^ at the dimeric interface (PDB ID: 6CT9). Mutated side-chains are indicated. The Zn^2+^-finger at the dimer interface is colored in magenta. (**G**, **H**) The NTase activities of 100 nM cGAS^FL^ variants under varying conditions. 250 μM ATP/GTP, with or without saturating 339-bp dsDNA and 5 mM MgCl_2_. 5 mM Mn^2+^ samples (blue) do not contain any MgCl_2_. Catalytically Dead: E225A/E227A ^3^. (**I**, **J**). IFN-1β-Luc reporter activities from HEK293T cells upon transfecting indicated amounts plasmids encoding cGAS^FL^ variants. *P < 0.05. Plasmids encoding STING (10 ng), Renilla Luciferase (5 ng), and IFN-1β-Firefly Luciferase (5 ng) were co-transfected in all samples. *n* = 3, ± SD. The luciferase activity with 50 ng WT cGAS^FL^ (**J**) is likely limited by the amount of the STING and/or IFN-1β-reporter plasmid.

The NTase activity of cGAS^FL^ without dsDNA was notably higher with Mn^2+^ (Figure 1B, dark blue), raising the possibility that Mn^2+^ alone activates the enzyme. Indeed, cGAS^FL^ showed robust dsDNA-free NTase activity with increasing Mn^2+^ (Figure 2A). The maximal activation without dsDNA was comparable to the dsDNA-induced activity (Figure 2A, Supplementary Table S6). The steady-state kinetic parameters of cGAS^FL^ with 5 mM Mn^2+^ in the absence of Mg^2+^ and dsDNA were also similar to those obtained in their presence (Figures 2B versus 1C; Supplementary Table S7). Furthermore, Mn^2+^-mediated, dsDNA-independent activation still occurred with excess Mg^2+^ (Figure 2C, Supplementary Table S6); 5 mM Mg^2+^ decreased the *k*_max_ by 2-fold and reduced the Hill constant, similar to when dsDNA was present (Figure 2A versus C, Supplementary Table S6). These observations consistently indicated that Mn^2+^ activates cGAS even without dsDNA. However, cGAS bound Mn^2+^ as much as 85-fold less tightly in the absence of dsDNA (EC_50_: 18 versus 1530 μM; Figure 2A and C; Supplementary Tables S5 and S6). Given that Mn^2+^ plus ATP/GTP enhances the dsDNA binding affinity of cGAS (Figure 1D), our results indicated that dsDNA-binding couples with Mn^2+^-utilization in a concerted manner. Collectively, we reveal an unexpected Mn^2+^-dependent, dsDNA-independent activation mechanism of cGAS. Moreover, our results indicate that dsDNA binding is crucial not only for allowing Mn^2+^ to be utilized at physiological concentrations, but also for synergizing with Mg^2+^.

### Mn^2+^ activates monomeric cGAS

We found previously that ATP/GTP binds cGAS even without dsDNA and modestly induces dimerization (19). Considering the positive relationship between dsDNA and Mn^2+^, we asked whether Mn^2+^ also promote substrate-mediated dimerization more efficiently than Mg^2+^. We thus monitored the changes in Förster resonance energy transfer (FRET) ratios between donor- and acceptor-labeled cGAS^FL^ populations with increasing Ap_c_pp/Gp_c_pp (non-hydrolyzable analogues that prevent turnover (19)). Ap_c_pp/Gp_c_pp produced higher FRET signals with Mn^2+^, suggesting that substrate-mediated dimerization is enhanced (Figure 2D). Nevertheless, FRET ratios did not change as robustly as those induced by 72-bp dsDNA (Figure 2D versus Supplementary Figure S2A), indicating that the dimeric fraction was still marginal. Moreover, unlike the NTase activity assays, FRET ratios did not change with increasing Mn^2+^ when Ap_c_pp/Gp_c_pp was fixed at a low concentration (Figure 2E; 200 μM, equivalent to [ATP/GTP] used in NTase assays in Figure 2A and C). These observations suggest that dimerization at this substrate concentration is minimal even with Mn^2+^. Adding Mn^2+^ and/or Ap_c_pp/Gp_c_pp did not alter dsDNA-induced FRET ratios, again indicating that dimerization is dictated by dsDNA (Supplementary Figure S2A). Nevertheless, the Mn^2+^-induced NTase activity of cGAS without dsDNA was as high as those induced by long dsDNA (Figure 2A–C), suggesting that Mn^2+^ activates monomeric cGAS (each monomer has an intact active-site (3,17,18,36,37)).

To test whether Mn^2+^ activates monomeric cGAS, we monitored the NTase activity of a mutant that cannot dimerize, K394E (Figure 2F, Figure S2B) (19). Both K394E-cGAS^FL^ and K394E-cGAS^cat^ displayed robust NTase activities with Mn^2+^ without dsDNA (Figure 2G, Supplementary Figure S2C–F), supporting the mechanism by which Mn^2+^ activates monomeric cGAS. Of note, cGAS has a Zn^2+^-finger adjacent to the dimer interface (Figure 2F), and mutating two coordinating Cys residues abrogates the signaling activity of cGAS in cells (17,36,37). Thus, to disrupt the dimerization in a different manner, we removed Zn^2+^ via EDTA or by mutation (C396A/C397A (36), Figure 2F). Unlike wild-type (WT) cGAS^FL^ that behaved a mixture of monomer and dimer in size-exclusion chromatography (SEC), both variants eluted as monomers (Supplementary Figure S2B), corroborating that the Zn^2+^-finger is important for dimerization. Both EDTA-treated and C396A/C397A-cGAS^FL^ variants bound dsDNA (Figure S2G); however, dsDNA failed to activate either variant with Mg^2+^ (Figure 2G). Nevertheless, both mutants showed robust NTase activities with Mn^2+^ in the absence of dsDNA or Mg^2+^ (Figure 2G). Our observations not only support the mechanism by which Mn^2+^ alone activates monomeric cGAS, but also indicate that Mn^2+^ does not substitute for Zn^2+^. Next, to explore biological relevance, we monitored the signaling activity of cGAS^FL^ variants with or without Mn^2+^ in HEK293T cells. A physiological amount of Mn^2+^ substantially increased the WT cGAS^FL^-STING induced IFN-β activity (Figure 2I). Mn^2+^ also induced IFN-β activity from K394E- and C396A/C397A-cGAS when they were sufficiently overexpressed (Figure 2I versus J); the dead variant showed no IFN-β activity in all conditions (Figure 2I-J). Together, these results suggest that Mn^2+^ activates monomeric cGAS.

### The positive coupling between Mn^2+^ and dsDNA occurs within the cGAS monomer

Dimerization is critical for the dsDNA-mediated activation of cGAS (15–17,19). Because our results suggest that Mn^2+^ activates monomeric cGAS, we then asked whether Mn^2+^ could restore dsDNA-mediated activation of cGAS mutants defective in dimerization (Figure 2F and G). Here, we determined the NTase activity of cGAS^FL^ mutants using a sub-optimal amount of Mn^2+^ for dsDNA-free activation of cGAS^FL^ (Figure 2H). As expected, all cGAS^FL^ variants, except for the catalytically dead, showed moderate Mn^2+^-induced NTase activity without dsDNA (Figure 2H light purple). Strikingly, dsDNA further stimulated the Mn^2+^-induced NTase activities of K394E, EDTA-treated, and C396A/397A variants (Figure 2H dark purple), which suggested that the positive coupling between Mn^2+^ and dsDNA occurs within a monomer (i.e. *in cis*). Next, to further test the *cis* coupling mechanism, we mutated two side-chains at the hinge of the ‘spine helix’ that changes conformation upon dsDNA binding (K173E/R176E (36), Figure 2F, Supplementary Figure S2H); the dsDNA-induced conformation change of the spine helix restructures the ‘activation loop’ *in cis* (3,18,36) (Supplementary Figure S2H). K173E/R176E-cGAS^FL^ migrated mostly as a dimer in SEC and bound dsDNA (Supplementary Figures S2B and G). 5 mM Mn^2+^ also activated K173E/R176E-cGAS^FL^ without dsDNA and Mg^2+^ (Figure 2G), but dsDNA failed to further activate this cGAS^FL^ mutant even with sub-saturating Mn^2+^ (Figure 2H). Surprisingly, however, K173E/R176E-cGAS showed robust IFN-β activity when overexpressed in HEK293T cells (Figure 2J). Considering that dsDNA does not activate this mutant (Figure 2H), our observation suggests that even the marginal catalytic activity of cGAS induced by Mn^2+^ alone could be sufficient for robust IFN-β signaling *in vivo*. Overall, our observations consistently indicate that the positive cooperativity between Mn^2+^ and dsDNA occurs within the cGAS monomer.

### Allosteric coupling between dsDNA and Mn^2+^ underpins substrate specificity

**Figure 3.**
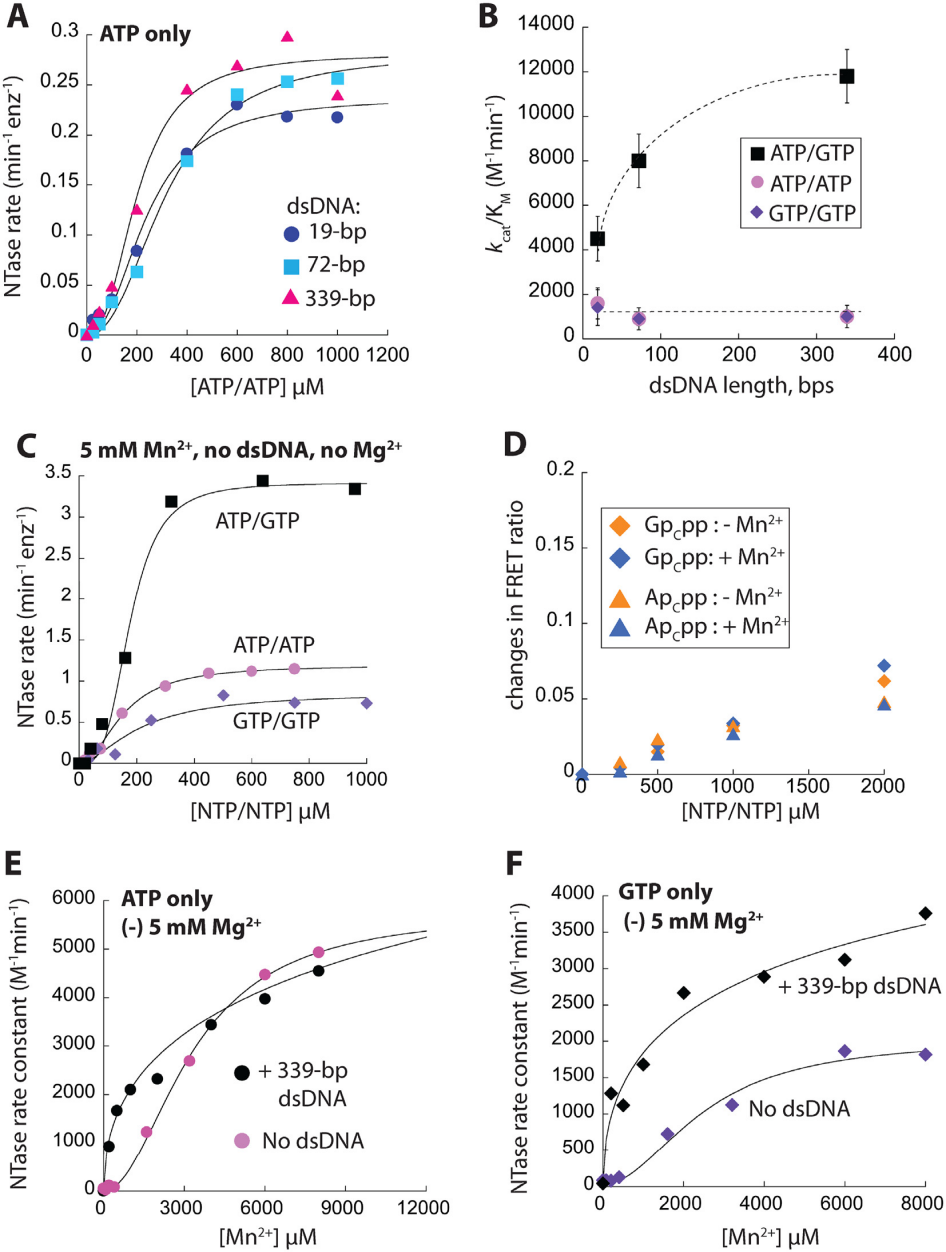
The positive coupling between Mn2+ and dsDNA binding requires cognate ATP/GTP. (**A**) The NTase activity of 100 nM cGAS^FL^ with increasing [ATP] and saturating amounts of various dsDNA lengths (5 mM MgCl_2_ and no MnCl_2_). (**B**) A plot of *k*_cat_/*K*_M_ for ATP/GTP, ATP/ATP, and GTP/GTP versus saturating amounts of different dsDNA lengths (5 mM MgCl_2_ and no MnCl_2_). Dotted lines trace the change in activity over increasing dsDNA lengths. *n* ≥ 3, ± SD. (**C**) The NTase activity of 100 nM cGAS^FL^ with increasing [NTP], 5 mM MnCl_2_, and without dsDNA or any MgCl_2_. (**D**) Changes in FRET ratios of 1:1 TAMRA:Cy5-labeled 20 nM cGAS^FL^ as a function of Ap_c_pp or Gp_c_pp. 5 mM MgCl_2_ and with or without 5 mM MnCl_2_. (**E**, **F**) The NTase activity of 100 nM cGAS^FL^ as a function of [MnCl_2_] without additional Mg^2+^ and in the presence or absence of saturating dsDNA. 200 μM NTP/NTP.

Although ATP/GTP is the cognate substrate pair, the active-site of cGAS is inherently promiscuous; cGAS can create linear dinucleotides using either ATP or GTP alone (pppApA and pppGpG, respectively) (3,21,22). This could be problematic *in vivo*, as ATP outnumbers GTP and neither pppApA nor pppGpG activates STING (3,21,22,26). Moreover, Mn^2+^ could exacerbate this issue, as it increases the substrate promiscuity of cGAS-like NTases (23–25). Nonetheless, cGAS can sustain the fidelity and efficiency of its signaling pathway. To understand the underlying mechanism, we first investigated how efficiently cGAS utilizes either ATP/ATP or GTP/GTP with Mg^2+^ and varying lengths of dsDNA using our NTase assay. With 19-bp dsDNA, both *k*_cat_s and *K*_M_s for noncognate substrates were ∼2-fold worse than those from ATP/GTP, resulting in ∼3-fold lower *k*_cat_/*K*_M_s (Figure 3A, B and Supplementary Table S8; see Supplementary Figure S3A for GTP/GTP). Our observations suggest that cGAS produces cGAMP and off-pathway dinucleotides with similar efficiency when bound to short dsDNA. Strikingly, and in contrast to ATP/GTP, longer dsDNA failed to enhance either *k*_cat_ or *K*_M_ against ATP/ATP or GTP/GTP, resulting in an up to 12-fold difference in *k*_cat_/*K*_M_ between cognate and noncognate substrates (Figure 3A and B, Supplementary Table S8). These results suggest an unexpected role of dsDNA length in governing the substrate specific catalytic activity of cGAS.

Mn^2+^-induced dsDNA-free NTase activity of cGAS^FL^ toward ATP/ATP or GTP/GTP was ∼3-fold higher compared those induced by dsDNA and Mg^2+^ (Figure 3C versus Figure 3A and Supplementary Figure S3A; see also Supplementary Table S8). Nevertheless, ATP/GTP still produced the highest Mn^2+^-induced *k*_cat_ (Figure 3C), which suggested that Mn^2+^ preserves the substrate preference of cGAS unlike several other related NTases (23–25). We then investigated whether ATP/ATP or GTP/GTP allows Mn^2+^ to synergize with dimerization/dsDNA-mediated activation. Here, we found that increasing Ap_c_pp or Gp_c_pp alone boosted the FRET ratios between two labeled cGAS^FL^ populations (Figure 3D), suggesting that binding of any NTP at the active-site promotes dimerization. However, Mn^2+^ did not further increase the FRET ratio for either NTP alone (Figure 3D versus 2D), suggesting that noncognate substrates do not allow Mn^2+^ to promote dimerization. Next, we asked how well cGAS utilizes Mn^2+^ in its NTase activity against noncognate pairs. Without dsDNA or excess Mg^2+^, the EC_50_ of Mn^2+^ against ATP/ATP or GTP/GTP was 2-fold weaker compared to ATP/GTP, but the Hill constants were still near two (Figures 2A versus 3E, F, Supplementary Figure S3B). Adding 5 mM Mg^2+^ in the absence of dsDNA moderately enhanced the EC_50_ of Mn^2+^ and reduced the Hill constant to near one (Supplementary Figure S3B-C and Table S9). However, with 339-bp dsDNA, the EC_50_ of Mn^2+^ against noncognate NTPs could not be determined due to apparent negative-cooperativity (Hill < 1; Figure 3E, F and Supplementary Figure S3B-C and Supplementary Table S9). These results suggest that dsDNA binding allosterically suppresses the utilization of Mn^2+^ in catalyzing noncognate substrates. Fixing the Hill constant to one for data fitting estimates the EC_50_s around 350 μM for both NTPs, which is 4-fold tighter than in the absence of dsDNA (Supplementary Table S9). Such changes pale in comparison to the 100-fold enhancement for ATP/GTP (EC_50_: 18 μM, Supplementary Table S5). Moreover, the low affinity would make the utilization of Mn^2+^ against noncognate substrates unlikely *in vivo* (20,30). Together, we concluded that positive coupling between dsDNA and Mn^2+^ requires cognate ATP/GTP.

### Mn^2+^ does not change product identity and accelerates both first and second linkage formation of cGAMP

**Figure 4.**
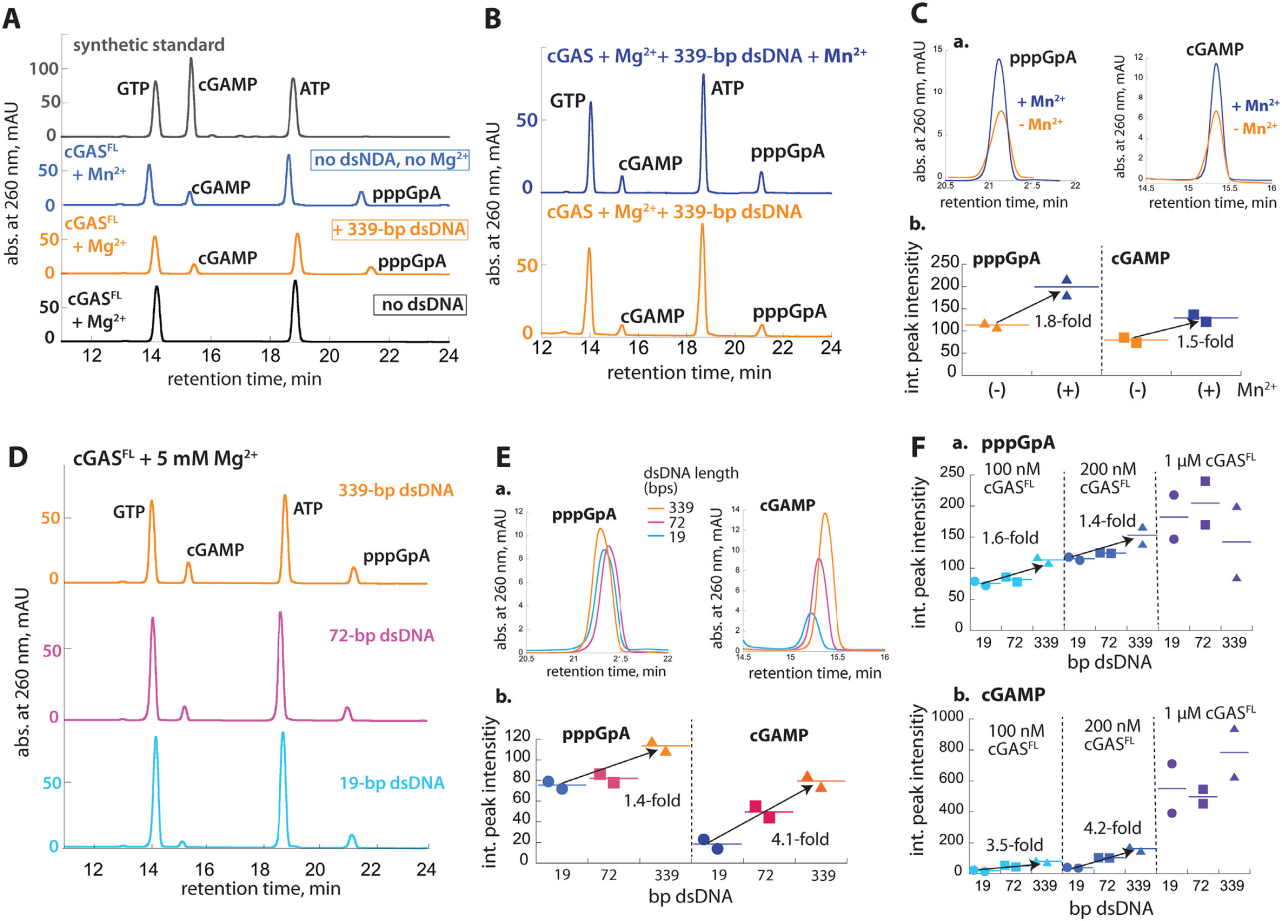
Mn^2+^ accelerates both pppGpA and cGAMP formation, and dsDNA length-dependent allostery governs the product fidelity. (**A**) HPLC traces of synthetic standards and cGAS^FL^ reaction products (two-hour reaction). 100 nM cGAS^FL^, 250 μM ATP/GTP, with or without saturating 339-bp dsDNA, and 5 mM MgCl_2_. The reaction with Mn^2+^ does not contain any MgCl_2_. (**B**) HPLC traces of cGAS^FL^ reaction products with or without 50 μM MnCl_2_ (two-hour reaction). 100 nM cGAS^FL^, 250 μM ATP/GTP, saturating 339-bp dsDNA, and 5 mM MgCl_2_. (**C**) a: Zoom-in of overlaid peaks corresponding to pppGpA and cGAMP from Figure 4B. b: A dot plot of the integrated (int.) peak intensities corresponding to pppGpA and cGAMP from experiments described in (B). Lines in all dot plots indicate the average of two independent experiments. (**D**) HPLC traces of cGAS^FL^ reaction products (two-hour reaction). 200 nM cGAS, 250 μM ATP/GTP, and saturating [dsDNA]. (**E**) a: Zoom-in of overlaid peaks corresponding to pppGpA and cGAMP from (D). b: A dot plots of the int. peak intensities corresponding to pppGpA and cGAMP from experiments described in Figure 4D. (**F**) Dot plots of int. peak intensities corresponding to pppGpA (a) and cGAMP (b) with various dsDNA lengths and cGAS^FL^ concentrations. 250 μM ATP/GTP and saturating [dsDNA].

cGAS first generates pppGpA from ATP/GTP then cyclizes the intermediate into cGAMP (3,22). It remained unclear which catalytic step was affected by Mn^2+^ and whether the same products were being produced. We thus analyzed various cGAS^FL^ reactions using High Pressured Liquid Chromatography (HPLC). We first established the retention times for ATP, GTP, and cGAMP using synthetic materials (Figure 4A, gray). With 5 mM Mg^2+^, we then confirmed that the peaks corresponding to cGAMP or pppGpA appeared only if dsDNA was present (Figure 4A, black versus orange). With saturating Mn^2+^, but without dsDNA or Mg^2+^, the same two peaks emerged (Figure 4A, blue), indicating that Mn^2+^ does not change the intrinsic cGAMP synthesis activity. dsDNA/Mg^2+^-induced cGAS reactions with or without 50 μM Mn^2+^ also produced the same peaks (Figure 4B). Notably, however, both cGAMP and pppGpA peaks were almost equally higher when Mn^2+^ was present (Figure 4B and C). These results suggest that either the first catalytic step, or both catalytic steps were accelerated. We thus concluded that Mn^2+^ potentiates the NTase activity of cGAS without altering product identity.

### The product fidelity is dictated by dsDNA length

Next, we asked whether dsDNA also accelerates both the first and second linkage formation in a length-dependent manner. Here, cGAS reaction products were identical across different dsDNA lengths (Figure 4D). However, while the peak corresponding to pppGpA was increased by 1.4-fold from 19- to 339-bp dsDNA, the cGAMP peak was 4.1-fold higher (Figure 4D and E), which are in stark contrast to when Mn^2+^ accelerated both steps equally (Figure 4C, b). Importantly, our results also suggest that dsDNA length allosterically dictates the rate-limiting cyclization. To further test this, we monitored reaction products resulting from different cGAS amounts, as promoting dimerization by raising protein concentrations decreases the dependence on dsDNA length in activating cGAS (19). pppGpA production increased linearly with increasing cGAS concentrations and dsDNA length-dependence was consistently marginal (Figure 4F and Supplementary Figure S4A–C). On the other hand, while cGAMP production depended on dsDNA length at lower cGAS concentrations, it became essentially length-independent at the highest concentration (Figure 4F, b, Supplementary Figure S4D-F), Our observations corroborate that dsDNA length-dependent dimerization is crucial for cyclization. Mn^2+^ did not alter this trend, but increased the amount of pppGpA and cGAMP for all dsDNA lengths even at the highest cGAS concentration (Supplementary Figure S4). Our observations again indicate that Mn^2+^ alone activates cGAS, and also synergizes with the allosteric activation. Overall, these results consistently suggest that dsDNA length-dependent dimerization regulates the cyclization of cGAMP from the intermediate.

### Allostery ensures the product fidelity even with excess ATP

**Figure 5.**
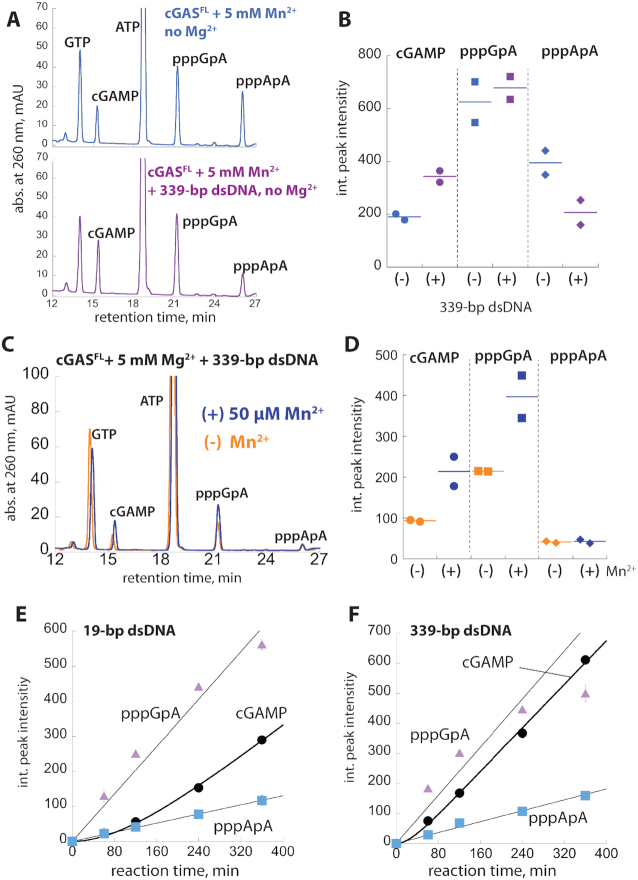
dsDNA length-dependent allostery dictates product fidelity of cGAS even with excess ATP. (**A**) HPLC traces of cGAS^FL^ reaction products (two-hour reaction) in the presence or absence of saturating 339-bp dsDNA without any MgCl_2_. 200 nM cGAS^FL^, 5 mM of MnCl_2_, 1.25 mM ATP and 0.25 mM GTP. (**B**) A dot plot of int. peak intensities for cGAMP, pppGpA and pppApA from experiments described in (A). (**C**) HPLC traces of cGAS^FL^ reaction products (two-hour reaction) in the presence of saturating dsDNA. 200 nM cGAS, 5 mM MgCl_2_, 1.25 mM ATP, 0.25 mM GTP and with or without 50 μM MnCl_2_. (**D**) A dot plot of int. peak intensities for cGAMP, pppGpA, and pppApA from experiments described in (C). (E, F) Kinetics of cGAMP, pppGpA and pppApA production with 19 (**E**) and 339-bp dsDNA (**F**). 200 nM cGAS^FL^, 5 mM MgCl_2_, saturating [dsDNA], 1.25 mM ATP and 0.25 mM GTP. Shown are averages of two independent experiments. The time-dependent cGAMP formation was fit as in (22). The time-dependent formations of pppGpA and pppApA were fit with linear equations.

The *in vivo* concentration of ATP is well above the *K*_M_ for cGAS and also present in ∼5-fold excess to GTP (27). Because Mn^2+^ boosts the NTase activity of cGAS against ATP/ATP (Figure 3), we envisioned that cytosolic Mn^2+^ might instigate futile catalytic cycles, accumulating an off-pathway product (pppApA). To test this possibility, we thus analyzed cGAS reaction products using physiologically relevant amounts of ATP:GTP (5ATP/1GTP). With 5 mM Mn^2+^, and without dsDNA or any Mg^2+^, we noted an additional peak indicating the production of pppApA along with cGAMP and pppGpA (Figure 5A, blue, see also Supplementary Figure S5A–C for ATP/ATP and GTP/GTP reactions). Adding 339-bp dsDNA did not change the pppGpA peak, but increased the cGAMP peak (Figure 5A, B, Supplementary Figure S5D). Remarkably, dsDNA also decreased pppApA production (Figure 5A, B, Supplementary Figure S5D), suggesting that the acceleration of cyclization by long dsDNA in turn suppresses off-pathway product formation. We also analyzed dsDNA/Mg^2+^-stimulated cGAS reaction with 5ATP/1GTP in the absence or presence of 50 μM Mn^2+^ (Figure 5C). Here, Mn^2+^ specifically increased the production of pppGpA and cGAMP without altering pppApA formation (Figure 5C, D, Supplementary Figure S5E), indicating that Mn^2+^ and longer dsDNA synergistically ensure the efficiency and fidelity of cGAS.

Finally, to further test the role of dsDNA length in suppressing off-pathway product formation, we monitored the kinetics of cGAS^FL^ reaction with 5ATP:1GTP in the presence of 19- or 339-bp dsDNA. In the presence of 19-bp dsDNA, both pppApA and pppGpA accumulated linearly, with pppGpA accumulating 5-fold faster than pppApA (Figure 5E, Supplementary Table S10). cGAMP production occurred with a prominent lag then reached a steady-steady (linear) rate (Figure 5E), corroborating the stepwise synthesis model (3,22). With 339-bp dsDNA, both pppGpA and pppApA accumulated about 1.3-fold faster than 19-bp dsDNA-induced production (Figure 5E versus F, Supplementary Table S10). However, the steady-state production of cGAMP was reached 4.3-fold faster than that induced by 19-bp dsDNA, quickly outpacing pppApA production (Figure 5E versus 5F, Supplementary Table S10). The negligible boost in pppApA formation by longer dsDNA also agrees with the dsDNA length-independent NTase activity of cGAS against ATP/ATP (Figure 3A). These results further support the mechanism by which dsDNA length-dependent allostery controls the signal fidelity of cGAS.

## DISCUSSION

**Figure 6.**
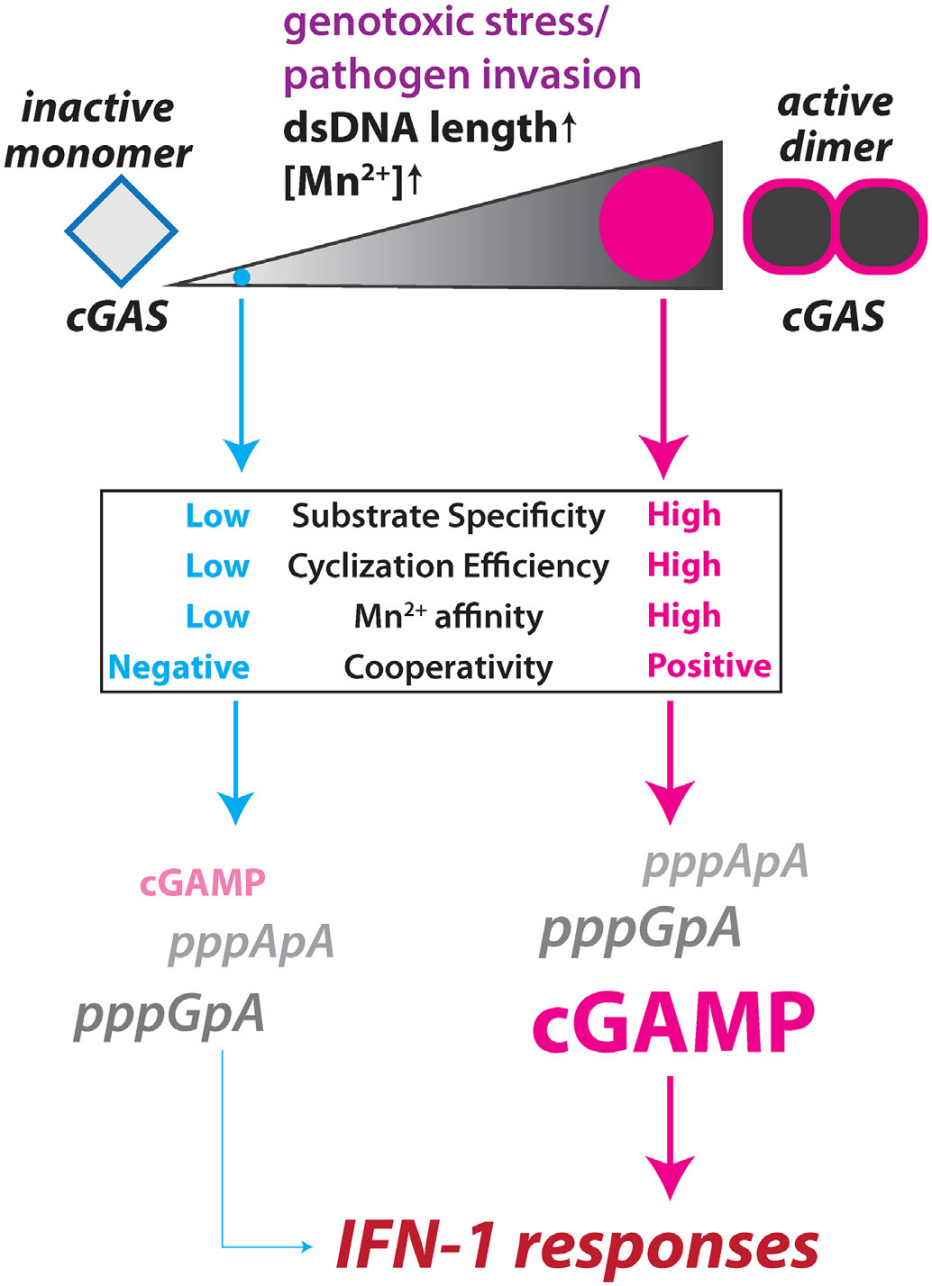
A scheme describing how allostery tunes the biochemical activities of cGAS With increasing stress levels (colored dots within the ramp), all signaling activities (substrate specificity, cyclization efficiency, Mn^2+^ utilization and cooperativity) are maximized, while the opposite occurs with decreasing stress levels. Such a dynamic mechanism would then lead to changes in product distribution, consequently tuning the signaling activity. Text sizes for dinucleotides indicate their relative abundance.

cGAS is central to the host defense against various maladies that give rise to cytosolic dsDNA (1). Despite extensive cellular and structural studies (3,15,18,19,28,36,37), how human cGAS operates remain poorly understood at the molecular level. For instance, how cytosolic Mn^2+^ released from damaged organelles potentiates cGAS activity remains unknown (20). Moreover, it is unclear how cGAS manages the inherent substrate promiscuity at its NTase active-site *in vivo* when its NTP substrates exist well above their respective *K*_M_s (Figure 3) with ATP present in large excess over GTP (27). Here, we identify an unexpected activation mechanism in which Mn^2+^ activates monomeric cGAS without dsDNA. More importantly, this noncanonical activation synergizes with the canonical dsDNA-dependent activation. Furthermore, we show that dsDNA length-dependent dimerization regulates the substrate specificity and cyclase activity of cGAS, with Mn^2+^ further polarizing these activities toward cognate ATP/GTP. Together, we propose that this uniquely synergetic coupling mechanism underpins the signaling efficiency, substrate specificity, and product fidelity of cGAS (Figure 6).

Cytosolic Mn^2+^ is crucial for the dsDNA sensing activity of cGAS (20). Unlike abundant Mg^2+^, Mn^2+^ is present in trace amounts and also sequestered in mitochondria and the Golgi (31,32,35). Pathogen invasion or exposure to toxic reagents damages these vital organelles, releasing Mn^2+^ to the cytosolic space (20). That is, the co-presence of Mn^2+^ and long dsDNA in the cytosol would signal one of the most critical conditions such as viral replication and/or massive mitochondrion deaths. Our work demonstrates the mechanisms by which cGAS senses and integrates multiple danger-associated molecular patterns within the environment that gives rise to cytosolic dsDNA, thus dialing its signaling outputs accordingly. The concentration of Mn^2+^ for dsDNA-free activation (EC_50_ ∼ 1 mM; Figure 2) is much higher than physiological levels (e.g. <10 μM in THP1 cells (20) and ∼50 μM in brain cells (30)); however, the affinity for Mn^2+^ drastically improves by dsDNA (Figure 1E and F). Such a highly cooperative mechanism would then ensure the host to properly initiate IFN-I responses.

The active-sites of cGAS-like NTases show varying degrees of promiscuity (23–25,28), and cGAS accepts different NTP pairs and generate off-pathway products (3,18,21,22,28). By contrast, human STING is hyper-selective towards cGAMP (3,21,22). cGAS is an unusually slow enzyme, as one round of cGAMP synthesis takes ∼20 s even under the best-case scenario (Figure 1C). Thus, if cGAS excessively generates off-pathway dinucleotides or fails to cyclize the on-pathway intermediate in a timely fashion would impinge on promptly mounting stress-responses. Further compounding issues include the imbalance between ATP and GTP (27), rate-limiting cyclization efficiency (3,8,22,28) and Mn^2+^ that often found to be mutagenic in other NTases (23–25). We find that the intrinsic allostery of cGAS solves these potentially graving problems. For instance, all biochemical activities required for efficient signaling are concertedly and selectively maximized with the correct signal, cognate substrates, and a specific metal cofactor (Figure 6). On the flip side, the same allosteric mechanism also permits greater promiscuity under normal, non-stressful conditions to minimize spurious activation (Figure 6). For example, unlike other NTases where Mn^2+^ favors noncognate NTPs (23–25), Mn^2+^ preferentially accelerates the *k*_cat_ of ATP/GTP for cGAS (Figure 3). Second, ATP/GTP is essential for the synergy between Mn^2+^ and dsDNA length-dependent activation (Figure 3). Third, dsDNA binding invokes anti-cooperativity in utilizing Mn^2+^ against either ATP or GTP alone, thereby further suppressing misincorporation of Mn^2+^ with noncognate substrates. Fourth, dsDNA length is irrelevant for the catalysis against ATP/ATP or GTP/GTP (Figure 3). Finally, longer dsDNA specifically accelerates the rate-limiting cyclization of cGAMP (Figures 3–5), which also suppresses off-pathway product formation even with Mn^2+^ and excess ATP (Figure 5). Considering that longer dsDNA promotes oligomerization (19) and subsequent liquid-phase condensation (LPC) of cGAS (38), it is tempting to speculate that phase-separated cGAS•dsDNA complexes trap the pppGpA intermediate to facilitate cyclization. Together, all these factors would synergistically maximize host innate immune responses against major intracellular assaults. On the other hand, short (or no) dsDNA decreases the affinity for Mn^2+^ (Figures 1 and 2), reduces the cyclization efficiency (Figures 4 and 5), and allows off-pathway reaction with noncognate NTPs to be almost as favorable as the on-pathway reaction with ATP/GTP (Figure 3B). All these actions would then synergistically minimize the signaling activity of cGAS (i.e. viral dsDNA has been degraded by host nucleases). Combined, such a tightly coupled allosteric mechanism would control the host innate immune system in a precise yet efficient manner.

Mg^2+^ is thought to be the canonical cofactor for cGAS-like NTases (23–25,39). However, a recent study revealed that Mn^2+^ could activate the catalytic efficiency of several bacterial cGAS-like NTases (39); the ligand requirements for these NTases remain largely unknown (39). Considering the highly concerted relationship between the dsDNA ligand and the Mn^2+^ cofactor we observe here, future mechanistic studies will reveal whether other NTases with signaling functions might entail similar ligand-cofactor selection mechanisms as cGAS. On the other hand, it is noteworthy that ‘short’ dsDNA (<40 bp) is relatively abundant in the cytoplasm (1,40,41). We found that a physiologically relevant amount of Mn^2+^ (50 μM) activated cGAS with minimal (or no) dsDNA when both wild-type and various mutant enzymes were overexpressed in HEK293T cells (Figure 2J). For instance, K173E/R176E-cGAS^FL^ did not respond to dsDNA *in vitro*, but showed robust activity in HEK293T cells with Mn^2+^ (Figure 2G versus J). Also importantly, the catalytic activity of these cGAS mutants were nonetheless marginal compared to wild-type (Figure 2H), indicating that cGAS does not have to operate at the maximal catalytic capacity to induce robust IFN-I signaling. Considering that IFN-I drives the overexpression of cGAS via a positive feedback loop (42), it is tempting to speculate that the Mn^2+^-induced activity of cGAS might underpin many type-I interferonopathies that do not involve pathogen infection (i.e. sterile inflammation), which include manganism (32), Parkinson's disease (43,44), and a wide range of autoimmune disorders such as Aicardi-Goutieres syndrome and systemic lupus erythematosus (7–10).

## Supplementary Material

gkaa084_Supplemental_Files

## Notes

Present address: Richard M. Hooy, Department of Molecular and Cell Biology, University of California, Berkeley, CA 94720, USA.
