# Supplementary material for: Allosteric coupling between Mn2+ and dsDNA controls the catalytic efficiency and fidelity of cGAS
Source: Nucleic Acids Res. 2020 Mar 14;48(8):4435–47. doi: 10.1093/nar/gkaa084 (PMC7192592; doi:10.1093/nar/gkaa084)

Supplementary Figure 1

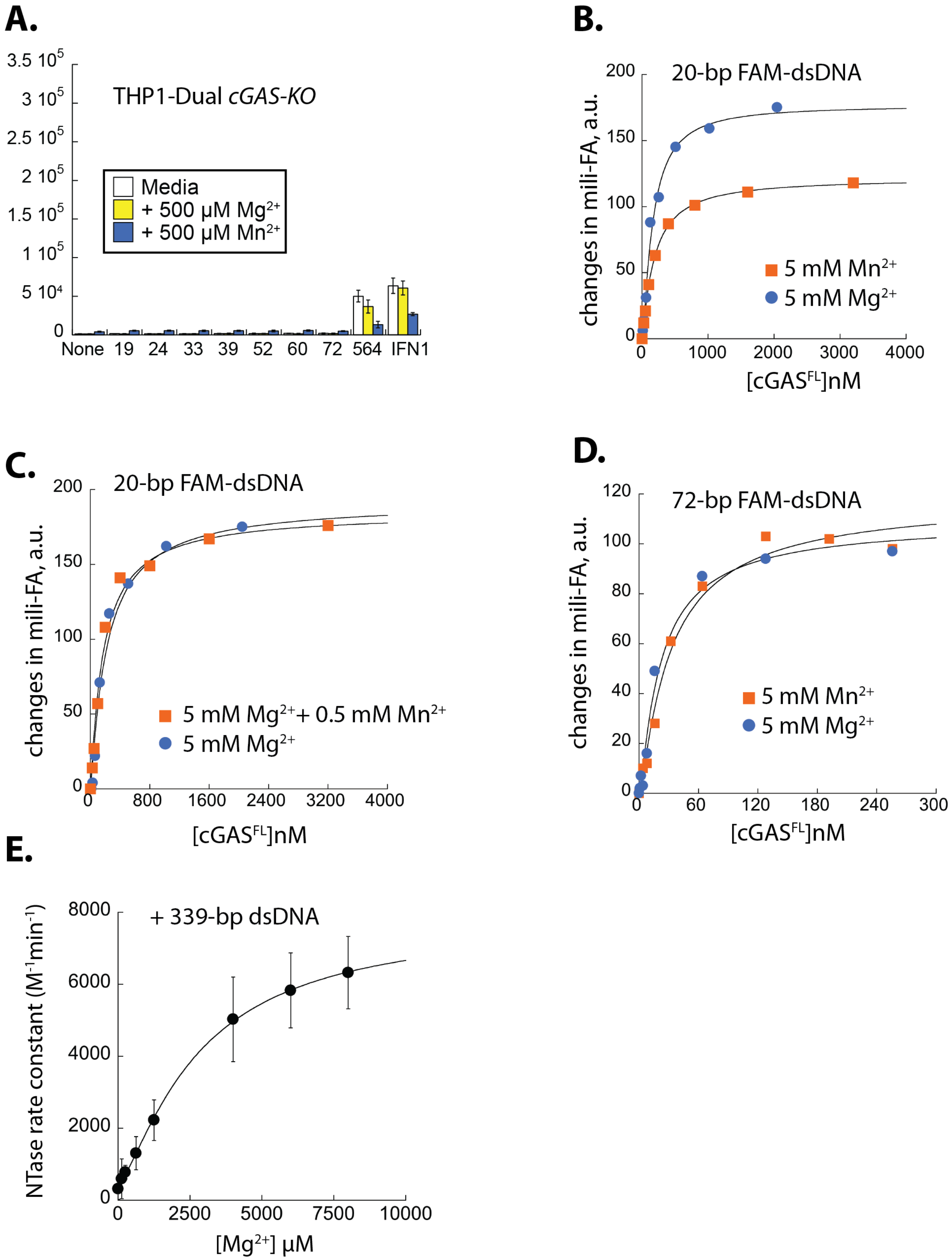

Supplementary Figure 2

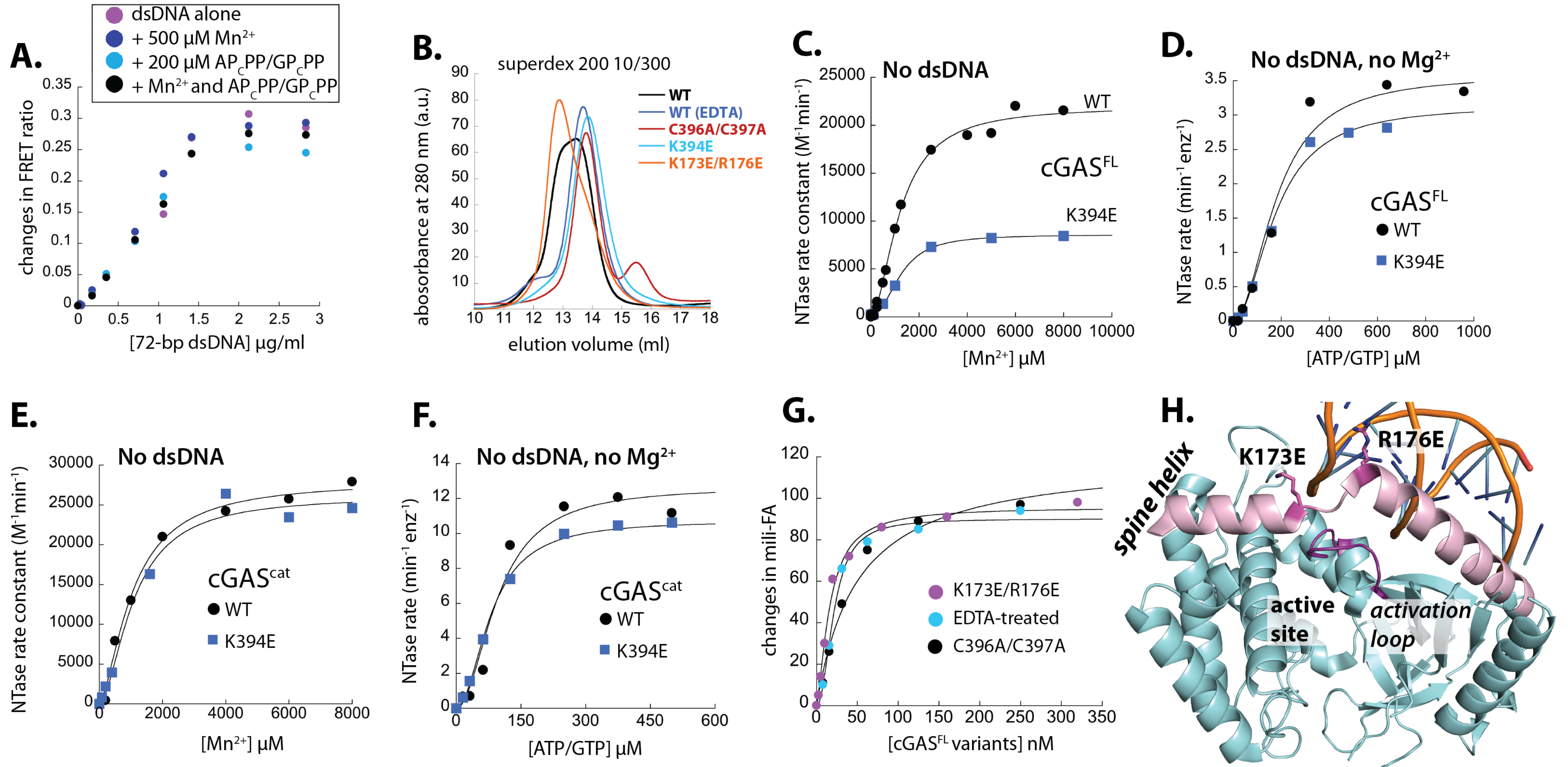

Supplementary Figure 3

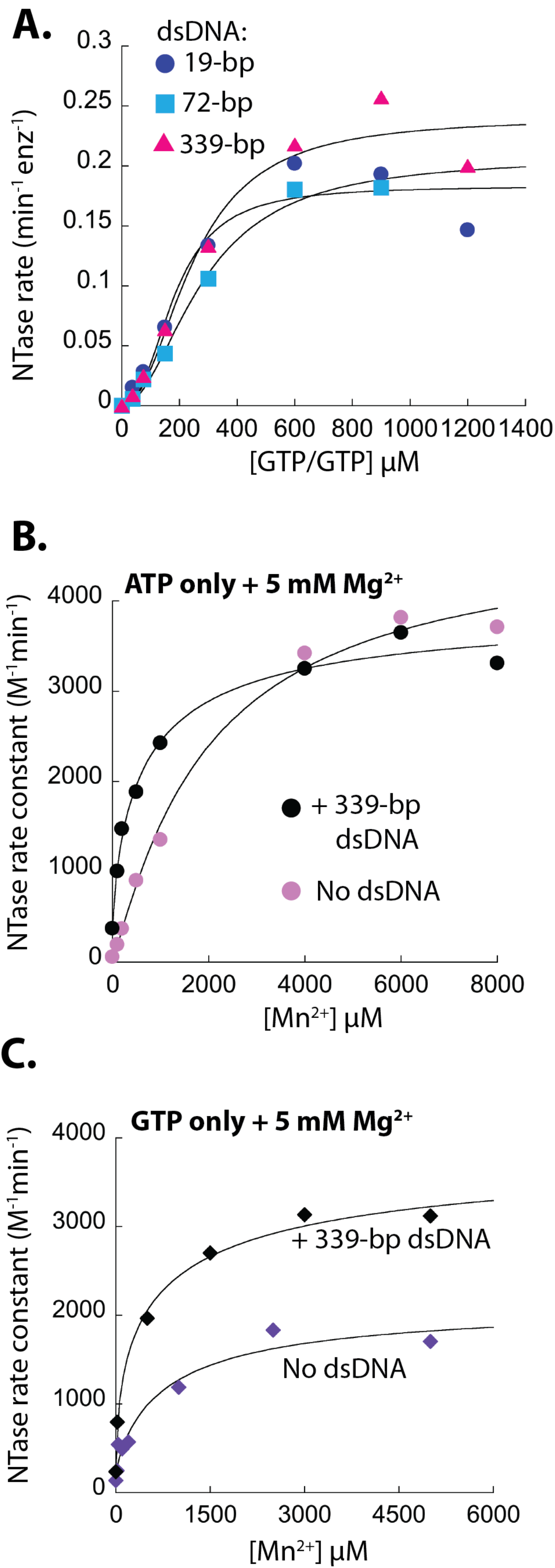

Supplementary Figure 4

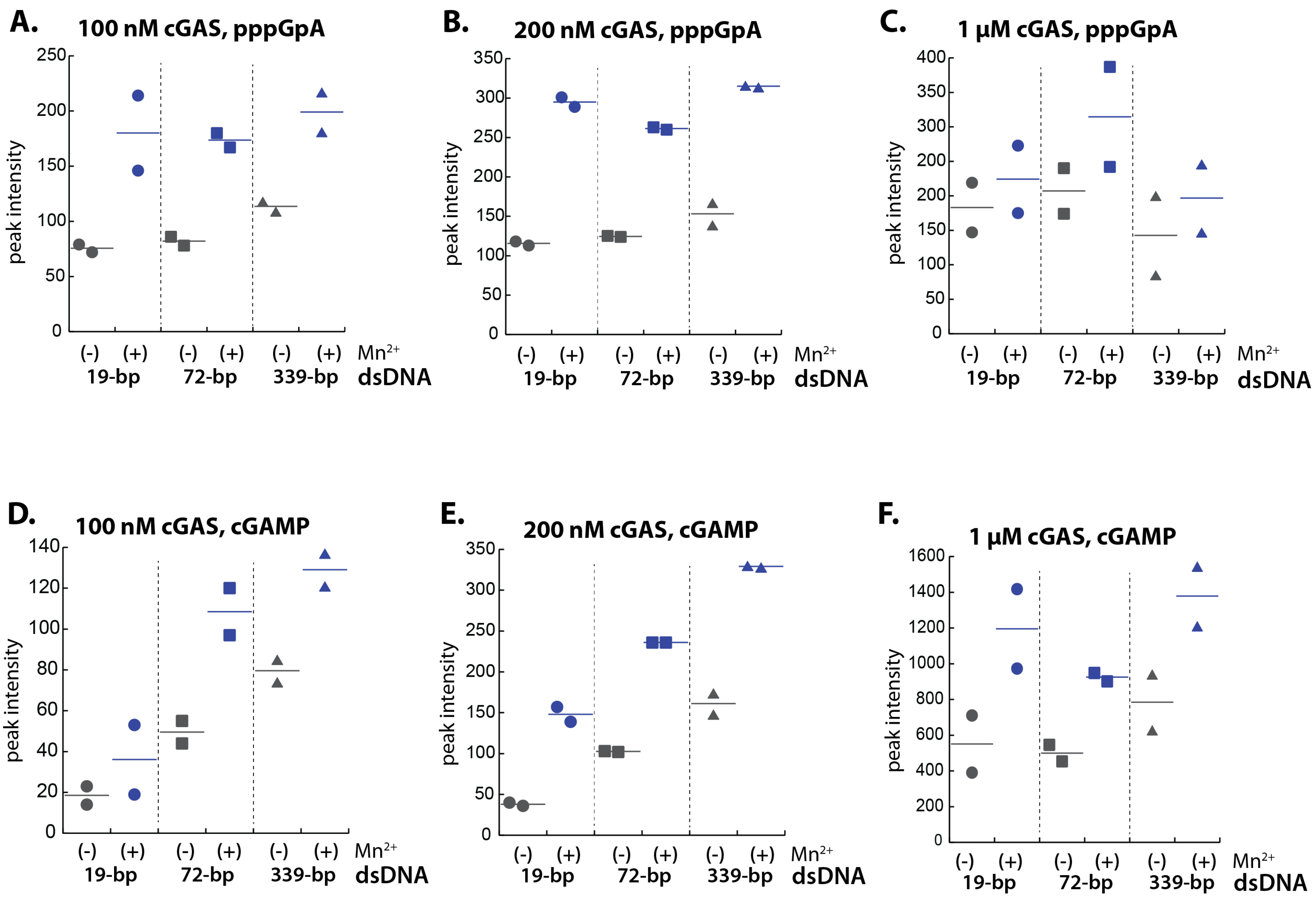

Supplementary Figure 5

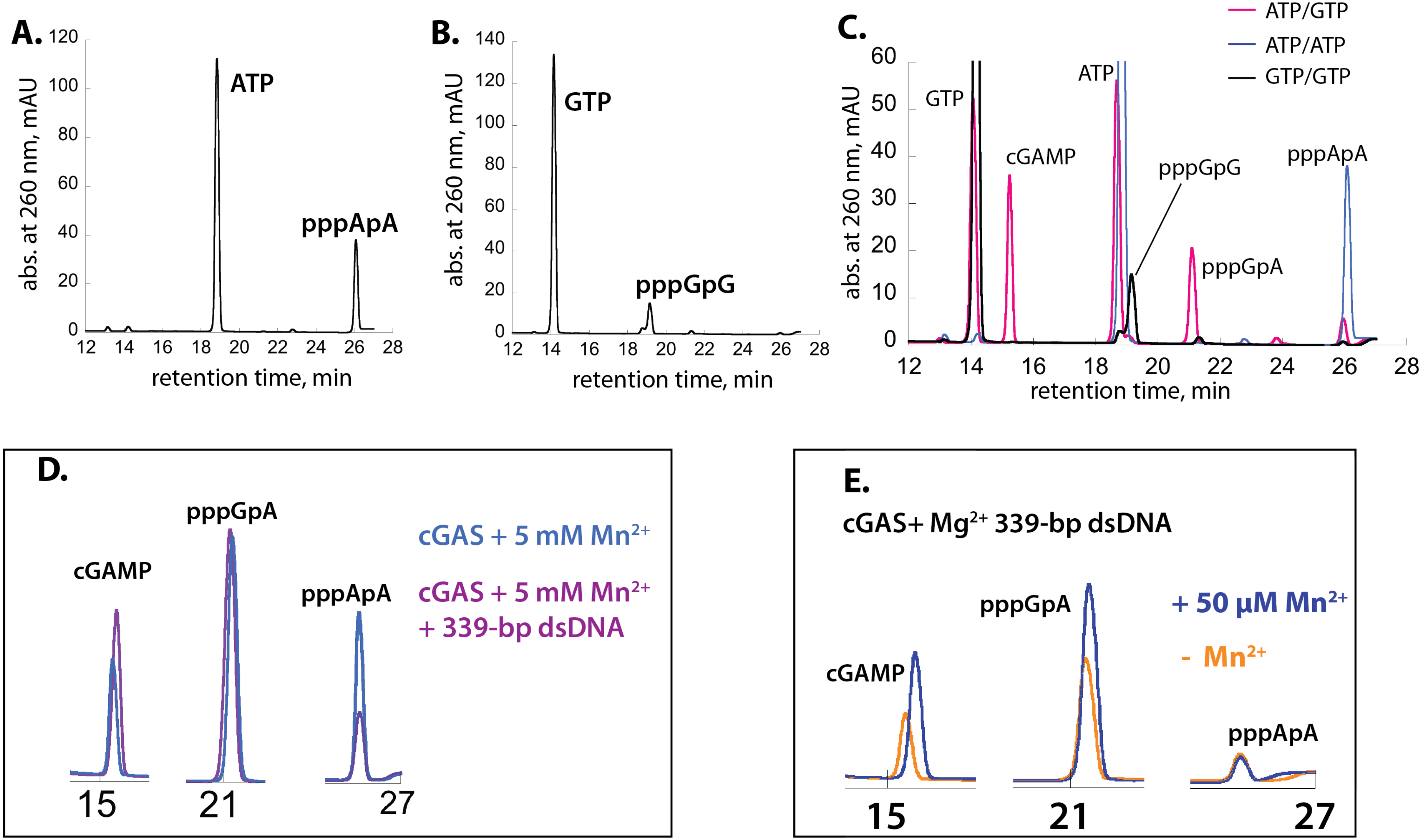

Supplement: gkaa084_Supplemental_Files [file gkaa084_supplemental_files.zip › HooyALL-SuppFigures 1-5.pdf]
