# Supplementary material for: Allosteric coupling between Mn2+ and dsDNA controls the catalytic efficiency and fidelity of cGAS
Source: Nucleic Acids Res. 2020 Mar 14;48(8):4435–47. doi: 10.1093/nar/gkaa084 (PMC7192592; doi:10.1093/nar/gkaa084)
Supplement: gkaa084_Supplemental_Files [file gkaa084_supplemental_files.zip › HooyALL-SuppTables.pdf]

Supplementary Tables

(1) Table summarizing dsDNA binding affinity of cGAS^{FL} in the presence or absence of Mn^{2+}. $n \geq 3$, ± SD.

	**5 mM $MgCl_2$**	**5 mM $MnCl_2$**	**5 mM $MgCl_2$ + 500 µM Mn^{2+}**
**dsDNA (bps)**	**K_D (µM)**	**K_D (µM)**	**K_D (µM)**
**19**	180 ± 20	150 ± 50	160 ± 4
**72**	26 ± 2	22 ± 3	

(2) Table summarizing steady-state kinetics parameters of cGAS^{FL} with saturating dsDNA. NTPs were pre-mixed with equimolar Mg^{2+}, except "None" (No dsDNA, No Mg^{2+}, 5 mM Mn^{2+}, and NTPs were also pre-mixed with equimolar Mn^{2+}). $n \geq 3$, ± SD.

	**5mM free Mg^{2+}, no Mn^{2+}**			
**dsDNA (bps)**	**k_{cat} (min^{-1} enz^{-1})**	**$K_{M,}$ ATP/GTP (µM)**	**Hill**	**k_{cat}/K_M ($M^{-1}min^{-1}$)**
**19**	0.75 ± 0.15	166 ± 13	2.1 ± 0.2	4500 ± 1000
**72**	1.20 ± 0.15	151 ± 13	2.4 ± 0.2	8000 ± 1200
**339**	1.31 ± 0.04	111 ± 11	2.0 ± 0.4	11800 ± 1200
	**5mM free Mg^{2+}+ 250 µM Mn^{2+}**			
**dsDNA (bps)**	**k_{cat} (min^{-1} enz^{-1})**	**$K_{M,}$ ATP/GTP (µM)**	**Hill**	**k_{cat}/K_M ($M^{-1}min^{-1}$)**
**19**	3.36 ± 0.23	154 ± 13	1.4 ± 0.2	21800 ± 2300
**72**	3.00 ± 0.47	76 ± 14	1.5 ± 0.1	39600 ± 9400
**339**	3.52 ± 0.74	103 ± 34	1.2 ± 0.1	34100 ± 13300

(3) Table summarizing [dsDNA] dependent activation of cGAS^{cat} ± Mn^{2+}. [ATP/GTP] = K_M for each dsDNA length. Reactions contain 5 mM $MgCl_2$ and NTPs were pre-mixed with equimolar Mg^{2+}. $n \geq 3$, ± SD

	**No Mn^{2+}**		**+ 250 µM Mn^{2+}**	
**dsDNA (bps)**	**k_{max} ($M^{-1}min^{-1}$)**	**K_{act} (µM)**	**k_{max} ($M^{-1}min^{-1}$)**	**K_{act} (µM)**
**19**	4200 ± 400	15 ± 5	40000 ± 15500	4.8 ± 1.4
**72**	4200 ± 1000	1.1 ± 0.4	22600 ± 1300	0.36 ± 0.03

(4) Table summarizing [Mg^{2+}] dependent activation of $cGAS^{FL}$. [ATP/GTP] = K_M 339-bp dsDNA. NTPs were pre-mixed with equimolar Mg^{2+}. $n \geq 3$, ± SD.

	**No free Mg^{2+}**		
**dsDNA (bps)**	**k_{max} ($M^{-1}min^{-1}$)**	**EC_{50} (μM)**	**Hill**
**339**	8900 ± 600	3900 ± 1400	1.2 ± 0.1

(5) Table summarizing [Mn^{2+}] dependent activation of $cGAS^{FL}$. [ATP/GTP] = K_M for each dsDNA length. NTPs were pre-mixed with equimolar Mg^{2+}. $n \geq 3$, ± SD.

	**No additional Mg^{2+}**		
**dsDNA (bps)**	**k_{max} ($M^{-1}min^{-1}$)**	**EC_{50} (μM)**	**Hill**
**19**	14400 ± 2300	100 ± 20	2.3 ± 0.4
**72**	12500 ± 1700	90 ± 20	1.4 ± 0.3
**339**	20600 ± 6700	43 ± 11	2.0 ± 0.2
	**(+) 5 mM Mg^{2+}**		
**dsDNA (bps)**	**k_{max} ($M^{-1}min^{-1}$)**	**EC_{50} (μM)**	**Hill**
**19**	12500 ± 1200	50 ± 20	1.0 ± 0.1
**72**	15700 ± 2900	30 ± 10	1.1 ± 0.1
**339**	10900 ± 1100	18 ± 3	1.0 ± 0.1

(6) Table summarizing [Mn^{2+}] dependent activation of $cGAS^{FL}$ without dsDNA. [ATP/GTP] = K_M for each dsDNA length. NTPs were pre-mixed with equimolar Mg^{2+}. $n \geq 3$, ± SD.

**No dsDNA**			
	**k_{max} ($M^{-1}min^{-1}$)**	**EC_{50} (μM)**	**Hill**
**No additional Mg^{2+}**	21300 ± 1800	1170 ± 150	1.9 ± 0.3
**(+) 5 mM Mg^{2+}**	12400 ± 2300	1530 ± 180	1.1 ± 0.1

(7) Table summarizing steady-state kinetics parameters of $cGAS^{FL}$ with saturating dsDNA. NTPs were also pre-mixed with equimolar Mn^{2+}, $n \geq 3$, ± SD.

	**No dsDNA, No Mg^{2+}**			
	**k_{cat} (min^{-1} enz^{-1})**	**K_M, ATP/GTP (μM)**	**Hill**	**k_{cat}/K_M ($M^{-1}min^{-1}$)**
**5 mM Mn^{2+}**	4.0 ± 0.6	190 ± 30	1.8 ± 0.5	21300 ± 4800

(8) Table summarizing steady-state kinetic parameters of $cGAS^{FL}$ with or without saturating dsDNA. Reactions without dsDNA ("None") contained 5 mM free Mn^{2+} and NTPs were pre-

mixed with equimolar Mn^{2+}. Reactions with dsDNA contained 5 mM free Mg^{2+} and NTPs were pre-mixed with equimolar Mg^{2+}. $n \geq 3$, ± SD.

	**ATP only**			
**dsDNA (bps)**	**k_{cat} (min^{-1} enz^{-1})**	**K_M, ATP/ATP (µM)**	**Hill**	**k_{cat}/K_M (M^{-1}min^{-1})**
**None (Mn^{2+})**	1.19 ± 0.00	82 ± 8	1.9 ± 0.3	14500 ± 1400
**19**	0.32 ± 0.11	200 ± 50	2.3 ± 0.1	1600 ± 700
**72**	0.24 ± 0.06	270 ± 60	2.4 ± 0.1	900 ± 300
**339**	0.26 ± 0.03	260 ± 80	1.9 ± 0.8	1000 ± 300
	**GTP only**			
**dsDNA (bps)**	**k_{cat} (min^{-1} enz^{-1})**	**K_M, GTP/GTP (µM)**	**Hill**	**k_{cat}/K_M (M^{-1}min^{-1})**
**None (Mn^{2+})**	0.92 ± 0.10	220 ± 70	1.7 ± 0.4	4100 ± 1400
**19**	0.45 ± 0.21	330 ± 100	1.7 ± 0.2	1400 ± 800
**72**	0.33 ± 0.16	360 ± 70	2.2 ± 0.2	900 ± 500
**339**	0.52 ± 0.22	490 ± 130	1.6 ± 0.1	1100 ± 500

(9) Table summarizing [Mn^{2+}] dependent activation of cGAS^{FL}. [ATP] and [GTP] = K_M. NTPs were pre-mixed with equimolar Mg^{2+}. $n \geq 3$, ± SD.

**No free Mg^{2+}**			
	**ATP only**		
**dsDNA (bps)**	**k_{max} (M^{-1}min^{-1})**	**EC_{50} (µM)**	**Hill**
**none**	5600 ± 300	2790 ± 840	1.8 ±0.2
**339**	9500 ± 8000	ND	0.8 ± 0.3
**339**	4500 ± 600	1440 ± 20	Fixed to 1
	**GTP only**		
**dsDNA (bps)**	**k_{max} (M^{-1}min^{-1})**	**EC_{50} (µM)**	**Hill**
**none**	2300 ± 300	2890 ± 520	1.9 ± 0.2
**339**	5900 ± 1200	ND	0.7 ± 0.3
**339**	4700 ± 1200	1040 ± 40	Fixed to 1
**5 mM Free Mg^{2+}**			
	**ATP only**		
**dsDNA (bps)**	**k_{max} (M^{-1}min^{-1})**	**EC_{50} (µM)**	**Hill**
**none**	4000 ± 1200	1500 ± 280	1.2 ± 0.2
**339**	4400 ± 700	ND	0.6 ± 0.1
**339**	3500 ± 300	360 ± 140	Fixed to 1
	**GTP only**		
**dsDNA (bps)**	**k_{max} (M^{-1}min^{-1})**	**EC_{50} (µM)**	**Hill**
**none**	3000 ± 900	1270 ± 320	0.9 ± 0.2
**339**	4800 ± 400	ND	0.5 ± 0.1
**339**	3700 ± 500	350 ± 90	Fixed to 1

ND: not determined; cannot be reliably determined due to anti-cooperativity

(10) Dinucleotide production rates (integrated peak intensity (a.u.)/ min).

dsDNA (bps)	pppApA	pppGpA	cGAMP (lag)	cGAMP (steady)
**19**	0.32	1.68	0.008	1.17
**339**	0.45	2*	0.036	1.81

* only first four points were used to generate linear fit
