# Supplementary material for: Allosteric coupling between Mn2+ and dsDNA controls the catalytic efficiency and fidelity of cGAS
Source: Nucleic Acids Res. 2020 Mar 14;48(8):4435–47. doi: 10.1093/nar/gkaa084 (PMC7192592; doi:10.1093/nar/gkaa084)
Supplement: gkaa084_Supplemental_Files [file gkaa084_supplemental_files.zip › SupFig LegendsR.docx]

**Supplementary Figure Legends**

**Supplementary Figure 1**

**(A)** dsDNA length-dependent signaling in THP-1 *cGAS-KO* dual cells in the presence of media, MgCl_2_ or MnCl_2_.

**(B-D)** Binding of cGAS^FL^ to fluorescein amidite (FAM)-labeled 20-bp or 72-bp dsDNA was monitored via tracking changes in fluorescence anisotropy (FA).

**(E)** The NTase activity of 25 nM cGAS^FL^ with increasing MgCl_2_. 250 µM ATP/GTP and saturating [dsDNA].

**Supplementary Figure 2**

**(A)** Changes in FRET ratios between 1:1 TAMRA:Cy5-labeled cGAS^FL^ (20 nM) with increasing 72-bp dsDNA under various conditions. 5 mM MgCl_2_ was included in all conditions.

**(B)** SEC profiles of purified cGAS^FL^ variants. WT and K173E/R176E show a mixture of monomer and dimer, while the other mutants migrate predominantly as monomers.

**(C)** The NTase activities of 100 nM cGAS^FL^ variants with increasing MnCl_2_ in the absence of dsDNA and 250 µM ATP/GTP.

**(D)** The NTase activities of 100 nM cGAS^FL^ variants with increasing ATP/GTP and 5 mM MnCl_2_. No dsDNA or MgCl_2_. ATP/GTP were also pre-complexed with Mn^2+^.

**(E)** The NTase activities of 100 nM cGAS^cat^ variants against 250 µM ATP/GTP with increasing MnCl_2_ in the absence of dsDNA.

**(F)** The NTase activities of 100 nM cGAS^cat^ variants with 5 mM MnCl_2_ and increasing ATP/GTP. No dsDNA or MgCl_2_. ATP/GTP were also pre-complexed with Mn^2+^.

**(G)**. Binding of cGAS^FL^ variants to FAM-labeled dsDNA-20bp was monitored by tracking changes in FA.

**(H).** Crystal structure of cGAS bound to dsDNA. The spine helix is colored in pink, and the activation loop in colored in magenta (PDB ID: 6CT9).

**Supplementary Figure 3**

**(A)** The NTase activity of 100 nM cGAS^FL^ with increasing [GTP] with saturating amounts of various dsDNA lengths.

**(B-C)** The NTase activity of cGAS^FL^ as a function of MnCl_2_ with or without dsDNA. 5 mM MgCl_2_, [ATP] and [GTP] = K_M_, [dsDNA] >> K_act_ (Hooy and Sohn, 2018).

**Supplementary Figure 4**

Dot plots of integrated peak intensities for pppGpA (**A-C**), and cGAMP (**D-F**) in the presence or absence of 50 µM MnCl_2_ with varying [cGAS^FL^] and dsDNA lengths.

**Supplementary Figure 5**

HPLC traces (two-hour reaction) of cGAS reaction products with 250 µM ATP only (**A**), and GTP only (**B**) with 5 mM Mn^2+^ and 200 nM cGAS in the absence of dsDNA. No MgCl_2_ was present and each NTP was pre-complexed with Mn^2+^. A single product from either nucleotide is consistent with the previous report that cGAS generates pppApA and pppGpG from ATP and GTP, respectively (Gao et al., 2013b; Hall et al., 2017)

**(C).** An overlay of cGAS reaction products from Supplemental Fig. 5A (ATP-only) and 5B (GTP-only), plus the reaction products from 200 nM cGAS^FL^ with 250 µM ATP/GTP and 5mM Mn^2+^. No MgCl_2_ was present and each NTP was pre-complexed with Mn^2+^.

**(D)**. Zoom-in of the HPLC trace from Figure 5A

**(E)**. Zoom-in of the HPLC trace from Figure 5C
